# Convalescent Plasma: The Relay Baton in the Race for Coronavirus Disease 2019 Treatment

**DOI:** 10.3389/fimmu.2020.570063

**Published:** 2020-09-23

**Authors:** Jing Ouyang, Stéphane Isnard, John Lin, Brandon Fombuena, Xiaorong Peng, Jean-Pierre Routy, Yaokai Chen

**Affiliations:** ^1^Infectious Diseases and Immunity in Global Health Program, Research Institute, McGill University Health Centre, Montréal, QC, Canada; ^2^Chronic Viral Illness Service, McGill University Health Centre, Montréal, QC, Canada; ^3^Chongqing Public Health Medical Center, Chongqing, China; ^4^Department of Microbiology and Immunology, McGill University, Montréal, QC, Canada; ^5^State Key Laboratory for Diagnosis and Treatment of Infectious Diseases, National Clinical Research Center for Infectious Diseases, Collaborative Innovation Center for Diagnosis and Treatment of Infectious Diseases, College of Medicine, The First Affiliated Hospital, Zhejiang University, Hangzhou, China; ^6^Division of Hematology, McGill University Health Centre, Montréal, QC, Canada

**Keywords:** coronavirus disease 2019, severe acute respiratory syndrome coronavirus-2, convalescent plasma therapy, immunotherapy, antibody

## Abstract

Coronavirus disease 2019 (COVID-19) is a pandemic caused by the severe acute respiratory syndrome coronavirus-2 (SARS-CoV-2). Most infected people have mild or moderate symptoms and recover without the need for extensive treatment. However, for seriously ill patients, no specific treatments are currently available. Convalescent plasma therapy (CPT), a passive immunotherapy, involves infusing plasma from recovered people into actively infected people, and is thought to be a specific intervention to improve outcome in patients with severe COVID-19. The presumed mechanism involves neutralizing antibodies and antibody dependent cytotoxicity/phagocytosis. Previous CPT trials showed an effect in SARS and pilot studies suggest CPT is an effective and safe strategy for seriously ill COVID-19 patients. CPT is currently being tested in large randomized clinical trials. Herein, we critically review the mechanism, applications and the challenges for CPT in the treatment of severe COVID-19, paving the way toward vaccine and immunotherapy development.

## Introduction

Since the first case reported in December 2019, the coronavirus disease 2019 (COVID-19), caused by the severe acute respiratory syndrome coronavirus-2 (SARS-CoV-2), has been declared as a pandemic (1). In a review by the WHO-China Joint Mission of 55,924 laboratory-confirmed cases in China, 80% of laboratory-confirmed patients with SARS-CoV-2 had mild disease and recovered, 13.8% had severe disease and 6.1% were critical (2). According to the WHO guidelines, supportive therapies are recommended to treat COVID-19 symptoms as specific treatments or vaccines for COVID-19 are currently unavailable, while many clinical trials are ongoing (3–10). Therefore, it is urgent to find effective treatment strategies for severe COVID-19.

Convalescent plasma therapy (CPT), a passive immunotherapy, involves infusing patients with antibody-rich plasma from people who have recovered from the same infection. As such, CPT has garnered attention in the treatment of severe COVID-19 as it immediately provides specific antibodies to control infection. Use of CPT can be traced back to the Nineteen century, when specific antibodies were sought from the serum of inoculated animals against diphtheria and tetanus (11). Since the 1900s, CPT has been used in the treatment of the Spanish flu, measles, arenaviruses, bunyaviruses, HIV, and coronaviruses (12–18). In 2014, WHO guidelines recommended convalescent plasma collected from patients recovered from Ebola disease for transfusion as an empirical treatment during outbreaks in Africa (19). Mair-Jenkins et al. (17) in 2015 further identified 32 studies of SARS-CoV infection and severe influenza revealing consistent evidences of a reduction in mortality, especially when convalescent plasma is administered early after symptom onset, compared with placebo or no therapy. Based on these study findings, the National Health Commission of China issued a Clinical Treatment Plan of Convalescent Plasma for COVID-19 in March 2020 (20). In April 2020, the US Food and Drug Administration in USA issued guidance for the administration and study of investigational convalescent plasma collected from individuals who have recovered from COVID-19 (21). Subsequently, several clinical trials on convalescent plasma for COVID-19 were initiated.

Herein, we critically review the potential for CPT to treat COVID-19, paving the way toward specific approaches with vaccine and immunotherapy development.

## Antigen-Specific Antibody Production and Viral Replication Inhibition

**Figure 1:**
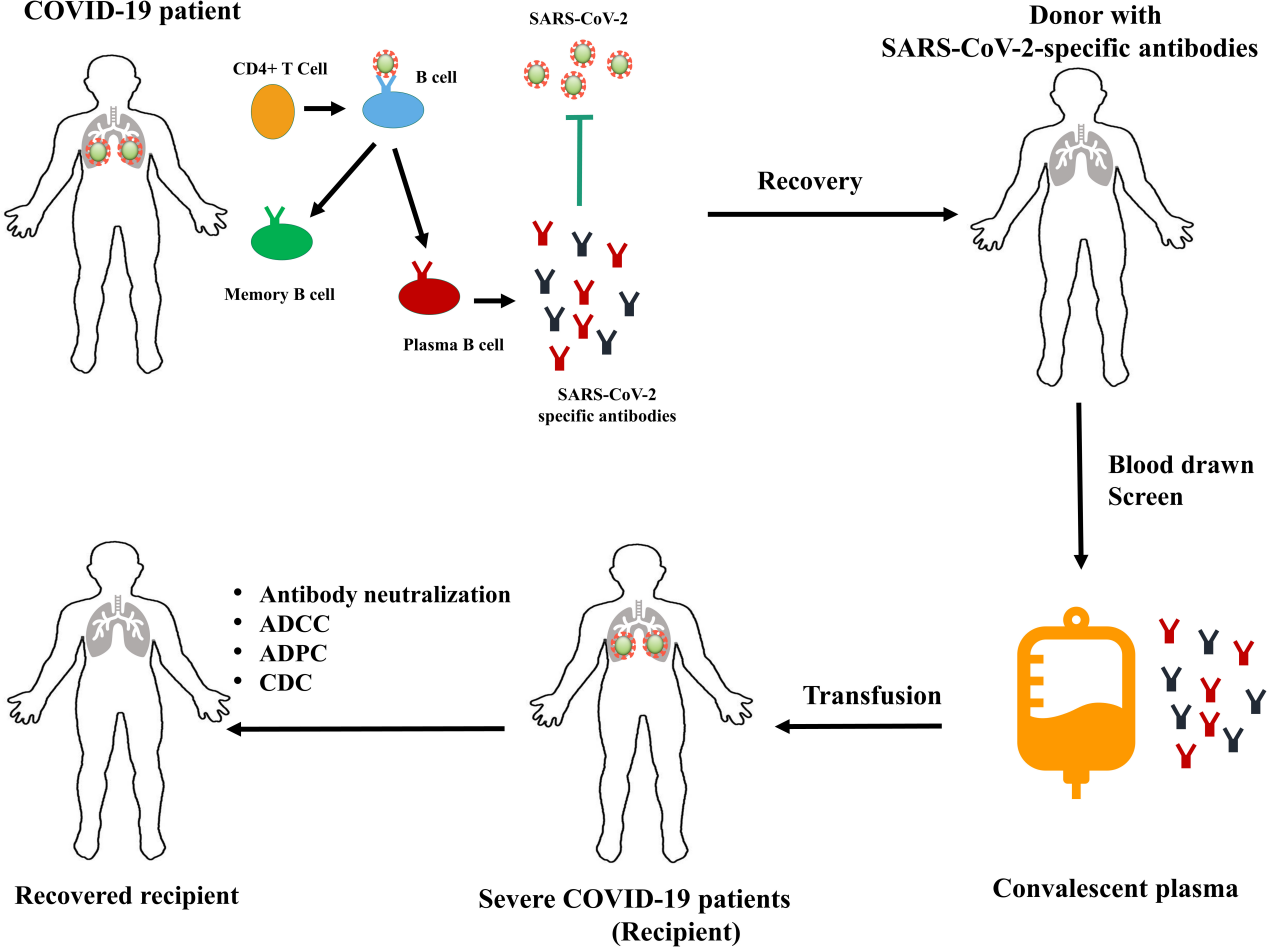
Schematic mechanism of convalescent plasma therapy for COVID-19. ADCC, antibody-dependent cellular cytotoxicity; ADCP, antibody-dependent cellular phagocytosis; CDC, complement-dependent cytotoxicity.

COVID-19 recovery and protection appears to be correlated with the development of anti-SARS-CoV-2 antibodies (22). As such, transfer of plasma antibodies from recovered patients could alleviate symptoms in severe cases in pilot studies. Schematic mechanism is shown in Figure 1.

Upon infection, viral antigens are disseminated to secondary lymphoid organs by antigen presenting cells and lymph flow. The antibody production process starts in germinal centers (GC) of lymph nodes that are highly organized: in the light zone, B cells encounter antigens, and follicular T cells (T_FH_) induce proliferation and favor survival of the antigen specific cells. In the dark zone, antigen-specific B cells start the antibody hypermutation and class switching processes, notably from immunoglobulin (Ig) M to IgG, in response to a context-specific microenvironment. Hypermutations allow the generation of new B-cells producing variations of antigen specific Ig. After migration back to the light zone, these newly generated B-cells will encounter T_FH_ that will favor another round of hypermutation leading to a higher affinity. These processes are further aided by T-cell help in the form of cytokines IL-4 and IL-21, and surface activation of CD40. Only B-cells with the highest affinity for the antigen will continue to divide and differentiate into antibody-producing cells (long-lived memory B cells), among which some will become plasmocytes, that will migrate into the bone marrow to allow long-term production of antibodies (23–25).

Different antibodies would induce various responses with specific functional outcome. Neutralizing antibodies with high affinity can recognize specific viral epitopes and block viral entry, fusion or egress (26). In the case of SARS-CoV, neutralizing antibodies can be raised directly against several SARS-CoV proteins, mainly targeting the spike protein which is involved in receptor recognition, virus attachment and cellular entry through angiotensin-converting enzyme 2 (ACE2) of the host (27–29). More recently, Chen et al. (30) showed that human monoclonal antibodies block the binding of SARS-CoV-2 spike protein to ACE2 receptor. Wu et al. (31) also reported that four monoclonal antibodies isolated from a COVID-19 convalescent patient displayed neutralization abilities by preventing binding of virus S-protein receptor binding domain (RBD) to ACE2.

Virus specific non-neutralizing antibodies may also influence outcome. All antibodies consist of an antigen-binding fragment (Fab) which binds to antigens, and a crystallizable fragment (Fc) that binds to cellular surface receptors and proteins from the complement system. The interaction with Fc-receptors can lead to virus-infected cell lysis through a variety of immune effector mechanisms, including antibody-dependent cellular cytotoxicity (ADCC) and antibody-dependent cellular phagocytosis (ADCP) (32). ADCC is a mechanism of cell-mediated immune defense whereby an effector cell of the immune system actively lyses a target cell, whose membrane-surface antigens have been bound by specific antibodies (33). ADCC classically involves natural killer (NK) or T- cells that interact with antigen-bound IgG and lyse the targeted cell (34, 35). ADCP is the uptake of virus-antibody complexes or antibody-coated virus-infected cells by phagocytic cells such as macrophages or neutrophils (32). In addition, antigen-bound antibodies may activate the complement system inducing complement-dependent cytotoxicity (CDC) (36). Thus, ADCC, ADCP and CDC may exert a broad range of immunomodulatory functions and contribute to the elimination of the virus.

Once a person recovers from a viral infection, antibodies will persist in blood to fight the same virus upon reinfection. SARS-CoV-specific antibodies were detectable about 2 weeks after infection, reaching a peak 60 days after and remaining elevated up to 180 days after (37). Concerningly, antibodies may not persist long-term in SARS patients, as virus-specific IgG titers peaked after 4 months and markedly decreased after one-year post-infection (38). Cao et al. (39) reported that SARS-CoV specific neutralizing antibodies declined gradually 4 months after infection, reaching undetectable levels in 16 % patients at month 36. In case of SARS-CoV-2, Long et al. (40) reported that all 285 patients with COVID-19 tested positive for antiviral immunoglobulin-G (IgG) within 19 days after symptom onset. Both IgG and IgM titers plateaued within 6 days after seroconversion. Wu et al. (41) reported that 70 % of 175 COVID-19 recovered patients on the day of discharge generated moderate to high levels of SARS-CoV-2 specific neutralizing antibodies in their plasma. COVID-19 patients generated SARS-CoV-2-specific neutralizing antibodies and spike-binding antibodies concurrently from day 10 to 15 after infection and remained stable. There is no obvious difference between the day of discharge and the subsequent visit in 2 weeks, indicating that SARS-CoV-2 specific-antibody levels are stable in the short term in convalescent donors. Chen et al. (30) successfully cloned two human blocking monoclonal antibodies using SARS-CoV-2 RBD-specific memory B cells isolated from recovered COVID-19 patients, indicating that antibodies can bind to the virus and prevent viral entry. Longer follow up studies are needed to decipher the dynamics of SARS-CoV-2-specific antibody production.

Following identification of those with high antibody titers and those with neutralizing function, convalescent plasma containing SARS-CoV-2-specific antibodies can be transfused into severely ill patients with COVID-19. These antibodies would recognize the virus and provide immediate antiviral effects. Animal models such as rhesus macaques can be used to further study the mechanism of these antibodies, as well as help determine plasma dose, treatment duration, efficacy and safety of CPT (42–44).

However, some studies suggest that severe cases have higher anti-SARS-CoV-2 IgG titers than non-severe cases (40), and antibodies might worsen disease outcome (26). In other SARS infections, high levels of anti-Spike protein promote pro-inflammatory responses of macrophages while decreasing their tissue repairability (45). As such, carefully designed clinical trials are needed to assess CPT influence on COVID-19 outcome.

## Application in COVID-19 Infection

Six studies, including 130 critically or severe ill patients with COVID-19 from China or Korea, were reported (Table 1). Pilot studies have shown potential protective effects of CPT in seriously ill patients (46–50). The clinical outcomes of all these patients have improved and they became negative for SARS-CoV-2 by PCR within 2–26 days after the transfusion of screened convalescent plasma. Additionally, no severe adverse effects were observed. However, these studies were uncontrolled case series without randomization nor control groups. As such, the clinical benefit of this intervention or whether patients might have spontaneously recovered without therapy is difficult to elucidate. In addition, aside from convalescent plasma, use of other antiviral and supportive therapies may induce bias and confound the direct contribution of convalescent plasma. Recently, in a randomized controlled trial (RCT), Li et al. (51) reported that CPT induced a significant clinical improvement within 28 days and a higher rate of negative PCR conversion in severe patients compared to a control group receiving standard treatment, but showed no significant effects in life-threatened patients.

**Table 1:** Reports of convalescent plasma in patients with COVID-19.

**References**	**Shen et al. (46)**	**Zhang et al. (47)**	**Duan et al. (48)**	**Ahn et al. (49)**	**Ye et al. (50)**	**Li et al. (51)**
**Country**	China	China	China	Korea	China	China
**Design**	Uncontrolled case series	Uncontrolled case series	Uncontrolled case series	Uncontrolled case series	Uncontrolled case series	RCT
**Clinical classification**	Critically ill patients	Critically ill patients	Severe patients	Severe patients	Critically ill patients	Severe or life-threatening patients
**Total number of participant (Male;female)**	5 (3, 2)	4 (2, 2)	10 (6, 4)	2 (1, 1)	6 (3, 3)	CPT group:52 (27, 25) Control group: 51 (33, 18)
**Age, year**	36–65	31–73	34–78	67–71	28–75	Median (IQR) CPT group 70 (62-80); Control group: 69 (63-76)
**Plasma volume (mL)**	400	200–2,400	200	500	200–600	4 to 13 mL/kg
**Antibody titer**	SARS-CoV-2–specific antibody (IgG) binding titer > 1:1,000 and neutralization titer > 40	Not determine	Neutralizing antibody titers above 1:640	Optical density ratio for SARS-CoV-2–specific antibody IgG > 0.5 (cut-off value 0.22).	Not mention	S-RBD–specific IgG titer of at least 1:640
**Other antiviral treatment**	2–3 antiviral agents	2–5 antiviral agents	1–3 antiviral agents	Lopinavir/ritonavir and hydroxychloroquine	Arbidol	CPT group: CPT+ Standard treatment in China; Control group: Standard treatment in China
**Efficacy**	(1) Body temperature normalized within 3 days in 4 of 5 patients. (2) Viral loads became negative within 12 days after the transfusion.	(1) Time from the transfusion to negative RT-PCR test results ranged from 3 to 22 days. (2) All the four patients recovered	(1) Clinical symptoms were significantly improved. (2) The viral load was undetectable after transfusion in all patients who had previous viremia with 2–6 days.	(1) Clinical symptoms were significantly improved. (2) SARS-CoV-2 was negative after 20 or 26 days.	(1) Clinical symptoms were significantly improved. (2) SARS-CoV-2 was negative after 8-12 days.	(1) For the clinical improvement within 28 days, CPT is significantly better than control in severe patient, but comparative in life-threatening patients. (2) No significant difference in 28-day mortality or time to discharge. (3) Significantly higher negative conversion rate of viral PCR in CPT group
**Safety**	Not reported	No associated serious adverse reactions	No severe adverse effects were observed.	No adverse reactions were observed.	No obvious adverse effect observed	Two patients in the CPT group experienced adverse events

*CPT, Convalescent plasma therapy; RCT, randomized controlled trial; RBD: receptor binding domain*.

These observations justify the need to assess efficacy and safety of CPT in well-designed clinical trials such as the ongoing studies proposed by investigators from Canada, China, Colombia, Iran, Italy and USA, presented in Table 2. These trials include observational case series and randomized controlled trials, with the size ranging from 10 to 1,200 individuals. For example, the CONvalescent Plasma for Hospitalized Adults With COVID-19 Respiratory Illness (CONCOR-1) (NCT04348656) will recruit 1,200 participants in a randomized open label trial. Johns Hopkins University are currently sponsoring a randomized, blinded phase 2 study (NCT04323800), which plans to include 150 participants to compare the efficacy and safety of SARS-CoV-2 convalescent plasma *vs*. control (SARS-CoV-2 non-immune plasma) among adults exposed to COVID-19.

**Table 2:** Ongoing clinical trials using convalescent plasma as a treatment for COVID-19.

**Clinical trial number**	**Aim**	**Number of participants**	**Intervention**	**Country**
NCT04292340	Treatment	15	Convalescent plasma	China
NCT04325672	Treatment	20	Convalescent plasma	United States
NCT04323800	Prophylaxis	150	Randomized groups ARM I: Anti-SARS-CoV-2 plasma ARM II: SARS-CoV-2 non-immune plasma	United States
NCT04332380	Treatment	10	Convalescent Plasma COVID-19	Colombia
NCT04340050	Treatment	10	Anti-SARS-CoV-2 convalescent plasma	United States
NCT04332835	Treatment	80	Randomized groups: ARM I: Anti-SARS-CoV-2 plasma+Azithromycin + Hydroxychloroquine ARM II: Azithromycin + Hydroxychloroquine	Colombia
NCT04327349	Treatment	30	Convalescent plasma	Iran
NCT04321421	Treatment	49	Hyperimmune plasma	Italy
NCT04333251	Treatment	115	Randomized groups ARM I: Anti-SARS-CoV-2 plasma ARM II: best supportive care	United States
NCT04348656	Treatment	1,200	ARM I: Anti-SARS-CoV-2 plasma ARM II: standard of care	Canada

## Challenges and Future Direction

RCT will allow the assessment of utility, efficacy and safety of CPT in COVID-19 patients. However, due to the diverse presentation of the disease, and taking into account the higher risks of developing severe COVID-19 in people with comorbidities, assessments of utility and safety should be evaluated in regard to participant's clinical characteristics and predispositions. Overall, ensuring the safety and efficacy of CPT remains a priority. At the same time, despite recent preliminary results of convalescent plasma for COVID-19, challenges in reducing risk and implementation in the clinic for patients with COVID-19 remain a concern.

There is a concern of antibody-dependent enhancement of infection (ADE), which occurs when antibodies facilitate viral entry into host cells and enhance viral infection (52, 53). ADE has been observed for a variety of viruses, such as flaviviruses, HIV and Ebola viruses (54–58). When patients are infected by one serotype of virus, they produce neutralizing antibodies targeting the same serotype of the virus. However, if they are later infected by a virus with a different serotype, the pre-existing antibodies cannot fully neutralize the virus. Instead, the antibodies first bind to the virus and then bind to the IgG Fc receptors on immune cells and mediate viral entry into these cells (59). The available evidence from the use of convalescent plasma in patients with SARS and MERS (17), suggest this does not happen when using plasma with high viral neutralization titers. Even so, it remains necessary to screen the viral serotype of donor and recipient to reduce risk of ADE. Furthermore, Crowe et al. reported that passively-acquired antibodies suppress humoral but not cell-mediated immunity in mice immunized with live attenuated respiratory syncytial virus vaccines (60). Therefore, CPT may attenuate immune responses leaving individuals vulnerable to subsequent re-infection.

Donor selection for convalescent plasma also poses a challenge. At the moment, screening donors for specific high-affinity neutralizing antibodies to SARS-CoV-2 may be difficult as there is no standardized assay for such measurement. However, both the FDA and Canadian Blood Services have cast a wide net for potential donors for convalescent plasma trials across North America. In the case of the FDA, convalescent plasma donors must meet the following criteria: (1) have a positive laboratory test through either a diagnostic nasopharyngeal swab or serological test for SARS-CoV-2 antibodies if the nasopharyngeal swab test was not performed, (2) be recovered from COVID-19 symptoms for a minimum of 14 days, with a negative nasopharyngeal swab test being unnecessary, and (3) have never been pregnant or have been tested negative for anti-HLA antibodies since their most recent pregnancy. Donors are recommended to have SARS-CoV-2 neutralizing antibody titers of at least 1:160 or 1:80, if titer measurements are available (61). Standardized tests assessing anti-SARS-CoV-2 titers and functions would prove useful in the global assessment of RCT results. Moreover, the influence of antibody titers and their antiviral function on clinical outcome should be tested in future clinical trials. In addition to usual appropriate testing for blood-born pathogens, the Canadian Blood Services and Hema-Quebec have implemented screening criteria for Canada's CONCOR-1 trial (NCT04348656), including being younger than 67 years of age, being confirmed positive through a laboratory test, and being fully recovered and symptom-free for a minimum of 28 days (62). As more data become available on the profiles of convalescent plasma donors, including neutralizing antibody titers, and as the supply of convalescent plasma units expands to cope with demand, the ability to whittle down and identify effective donors will also be refined.

Although US and China have issued guidelines for the implementation of CPT clinically (63), there are still gaps needed to be explored, such as the plasma dose, treatment duration, antibody stability and preservation. Historically, the dosing of convalescent plasma has been highly variable for different viruses. The total dose of the patients from the reported COVID-19 studies also ranges from 200 to 2,400 mL (46–49). Therefore, the dose-effect and time-effect relationships require further study.

Most clinical trials focus on reactive treatment after the onset of symptoms, although previous experience with CPT against SARS or influenza infections showed most benefits before the onset of symptoms. However, detecting and treating people before the onset of symptoms represent a logistical challenge. Moreover, severe cases appear to have higher levels of anti-SARS-CoV-2 IgG, hampering the hypothesis of antibodies preventing COVID-19 severity (40). Hence post-exposure prophylaxis might be a particular situation where CPT might decrease COVID-19 clinical outcome (NCT04323800).

Moreover, beyond the ABO plasma compatibility, CPT can result in transfusion-related lung injury and transfusion-related circulatory fluid overload, especially in the predominantly elderly population of those affected by COVID-19. Close medical and nursing surveillance are needed in hospital setting, limiting the scaling-up of such intervention (64).

## Conclusion

COVID-19 is a new global health crisis currently lacking specific treatments or vaccines. CPT has been explored in other infections and its efficacy and safety in the treatment of COVID-19 is currently being assessed in several clinical trials, including large RCT.

Determining clinical markers of appropriate timing and dose of CPT is the current priority before implementation in the clinical setting. In the future, the dynamics and mechanism of anti-SARS-CoV-2 specific antibody and antibody-dependent effects need to be studied at the cellular level with specific T and B cell responses to drive further innovation in treatment. Overall, collaborative efforts encompassing immunology, hematology, clinical care and pharmacy will optimize and facilitate CPT in the fight against COVID-19, paving the way for targeted therapies and vaccines.

## Author Contributions

JO, SI, and JL wrote the first draft of the manuscript. BF and XP provided critical revision of the manuscript. J-PR and YC conceived and designed the manuscript. All authors approved it for publication.

## Conflict of Interest

The authors declare that the research was conducted in the absence of any commercial or financial relationships that could be construed as a potential conflict of interest.
